# Population-level trends over a decade in geographical inequality for opportunity in access to maternal care services: a cross-sectional analysis from the National Family Health Surveys in India

**DOI:** 10.1136/bmjopen-2024-083922

**Published:** 2024-11-21

**Authors:** Rakhi Dandona, Moutushi Majumder, G Anil Kumar

**Affiliations:** 1 Public Health Foundation of India, New Delhi, India; 2 Institute for Health Metrics and Evaluation, University of Washington, Seattle, Washington, USA

**Keywords:** Pregnant Women, Primary Health Care, PUBLIC HEALTH, Surveys and Questionnaires, Health Equity, Health Services, Antenatal care services, continuum of care, human opportunity index, India, inequity, institutional delivery, NFHS, postnatal care, wealth index

## Abstract

**Abstract:**

**Objectives:**

The objective of this study is to examine the trends in geographical inequality of opportunity in maternal health services in India considering the Every Newborn Action Plan (ENAP) 2025 coverage targets.

**Setting:**

India.

**Participants:**

Women in the National Family Health Survey (NFHS)—NFHS-4 (2014–2015) and NFHS-5 (2019–2021).

**Primary and secondary measures:**

District-level coverages of 4+antenatal care (ANC) visits, institutional delivery with skilled birth attendant, postnatal care (PNC) services within 48 hours of delivery, continuum of care (CoC) services for women with most recent live births were considered. Human Opportunity Index (HOI) documented the opportunities for equitable access to these services, ranging from 0 (high inequality) to 100 (universal access). HOI was compared between the survey rounds and wealth index (WI) quintiles that the women belonged to.

**Results:**

Coverages of 4+ANC visits, institutional delivery, PNC and CoC in India increased by 22.8% (95% CI 22.1% to 23.5%), 12.6% (95% CI 12.2% to 12.9%), 28.5% (95% CI 28.0% to 29.0%) and 38.6% (95% CI 37.6% to 39.6%) between NFHS-4 and NFHS-5, respectively. The HOI for 4+ANC visits was 48.4 in NFHS-5, ranging from 11.3 to 92.4 in states and from 31.1 to 70.5 for WI I–V. The HOI for institutional delivery was 80.4 in NFHS-5, ranging from 37.4 to 99.7 in the states and from 21.0 to 100 for WI I–V. The HOI for PNC services was 73.5 in NFHS-5, ranging from 37.5 to 95.6 in the states and from 61.2 to 88.3 for WI I–V. The HOI for CoC was 37.1, ranging from 6.5 to 88.8 in the states and from 19.8 to 62.7 for WI I–V for CoC in NFHS-5.

**Conclusion:**

Though significant improvements in the geographical inequity of maternal health services have been made in India, the geographical inequity for 4+ANC visits coverage lags significantly behind resulting in CoC coverage inequity to achieve the ENAP targets for these services.

**STRENGTHS AND LIMITATIONS OF THIS STUDY**

- District-level inequity estimated in the coverage of maternal health services for India using large-scale demographic surveys.
- Human Opportunity Index is used to measure not only the distribution of coverage of maternal health services but also how fairly the available services are distributed among women by geography and by wealth index.
- Socioeconomic inequities in maternal health services in India have been reported previously but the use of Human Opportunity Index to measure inequity has not been attempted.
- Quality of services was not captured in the assessment.

## Introduction

 With the Sustainable Development Goal (SDG) 10, governments worldwide have committed to act on inequality through multiple, interconnected goals, requiring combined policy action in order to meet an overall commitment to ‘leave no-one behind’.^1^ Access to healthcare for all also intersects with the inequalities related to gender, socio-economic status, education levels, employment status and geographical location, with the most marginalised being the least able to access quality healthcare.^2^ Inequality in access to essential healthcare services has implications for achieving universal health coverage, including maternal health services among both the general and the most disadvantaged populations.^3^ Significant literature on the extent of inequities in maternal health services is available from developing countries including from India, predominately based on the Demographic and Health Surveys (DHS) and focusing on socioeconomic inequalities in the use of maternal health services.^4^,^16^

In the context of Countdown to 2030, SDGs 3.1 and 3.2 which aim to reduce maternal and neonatal mortality by 2030,^3^ we examine the trends in geographical inequality of opportunity in access for the coverage of antenatal care (ANC) visits, institutional delivery with skilled birth attendant (SBA) and postnatal care (PNC) visits within 48 hours in India using the National Family Health Survey (NFHS), which is the equivalent of DHS in India.^17^ In addition, we also measure geographical inequality in the coverage of continuum of care (CoC), which is recommended as one of the global strategies for maternal health to improve maternal and neonatal outcomes in the developing country setting.^18^,^20^ The maternal health programme in India is implemented at the district level,^21^ and the Every Newborn Action Plan (ENAP) 2025 provides for maternal care services coverage target indicators based on the district-level coverage of these services in a state.^22^ Therefore, we examine to define geographical inequality at the district level in India and its states as these have implications on planning to achieve the ENAP 2025 coverage targets.

## Methods

We used the publicly available data from the two most recent rounds of NFHS conducted post year 2010, NFHS-4 (2014–2015) and NFHS-5 (2019–2021).^17^ ^23^ These nationally representative population-based surveys are conducted by the International Institute of Population Sciences, Mumbai, India, with the primary aim to provide estimates of maternal and child health and reproductive health indicators at the district level for India.^17^ ^23^

The NFHS documents data on the most recent live birth for ever-married women in the last 5 years from the time of data collection. The NFHS-4 documented data for live births born between 2011 and early 2016 whereas the NFHS-5 documented data on livebirths born between 2016 and early 2021. We defined the CoC for this analysis as a woman having reported four or more ANC visits and institutional delivery with SBA and PNC within 48 hours of delivery for her most recent livebirth in the last 5 years. We calculated the coverage of 4 or more ANC visits (4+ANC visits), institutional delivery with SBA, PNC within 48 hours of delivery and CoC for the most recent live birth for NFHS-4 and NFHS-5 for India, and its states and districts.

### Data analysis

We used the Human Opportunity Index (HOI) to measure the trends in geographical inequality for the opportunity to access maternal care services in India and its states. The HOI is a measure of the coverage rate of an opportunity discounted by inequality in the distribution across the circumstance groups, where D measures the dissimilarity between access to services for groups defined by circumstance characteristics (such as wealth index, education, distanceetc) and the average access coverage for the same service for the population as a whole; C is average access coverage of services the population as a whole and is estimated as^24 25^:

*HOI*=(1*−D*)*×C*

The HOI value ranges from 0 (high inequality) to 100 (universal access). In this analysis, D is the index of geographical inequality at the district level within each state compared with the average coverage of a particular maternal service for a given state. The HOI for coverage of each of the three maternal care services and CoC for India and its states was estimated using the district level coverage of these services in NFHS-4 and NFHS-5, and the change in HOI between NFHS-4 and NFHS-5 was assessed. The number of districts in each state in NFHS-4 and NFHS-5 is shown in online supplemental table 1.

In addition, we explored the geographical inequality within each of the wealth index (WI) quintile in NFHS-5. The WI quintile is provided in the NFHS dataset at the household level which is based on the number and kinds of consumer goods each household owns calculated using principal component analysis.^17^ ^26^ We calculated HOI for India and states for each WI quintile for each maternal health service and CoC. We report the ratio of state average HOI with WI I and WI V, and the ratio of HOI WI I to WI V for each maternal health service and CoC.

The states of India were grouped based on their development status for this analysis. The Empowered Action Group of states as categorised by the government of India (Bihar, Chhattisgarh, Jharkhand, Madhya Pradesh, Odisha, Rajasthan, Uttar Pradesh, Uttarakhand and the north-eastern states (Arunachal Pradesh, Assam, Manipur, Meghalaya, Mizoram, Nagaland, Sikkim and Tripura) were grouped as ‘less developed’ and the remaining states were categorised as ‘more developed’.^27 28^ Delhi, Jammu and Kashmir including Ladakh were considered as a state for this analysis. The other Union Territories were excluded as there are no districts in these Union Territories to undertake this analysis. We have reported 95% confidence interval for all estimates as relevant and all the analyses were carried out using STATA V.13.1 and Microsoft Excel 2016.

### Patient and public involvement

Patients were not involved in planning of this analysis.

## Results

A total of 188 506 ever-married women provided data on 249 020 live births in NFHS-4 and 174 796 ever-married women provided data on 195 277 live births in NFHS-5. The coverages for 4+ANC visits, institutional delivery with SBA, and PNC in 48 hours, and CoC for NFHS-4 and NFHS-5 for India, grouping of states, and individual states are shown in online supplemental table 2.

### 4+ANC visits

**Figure 1:**
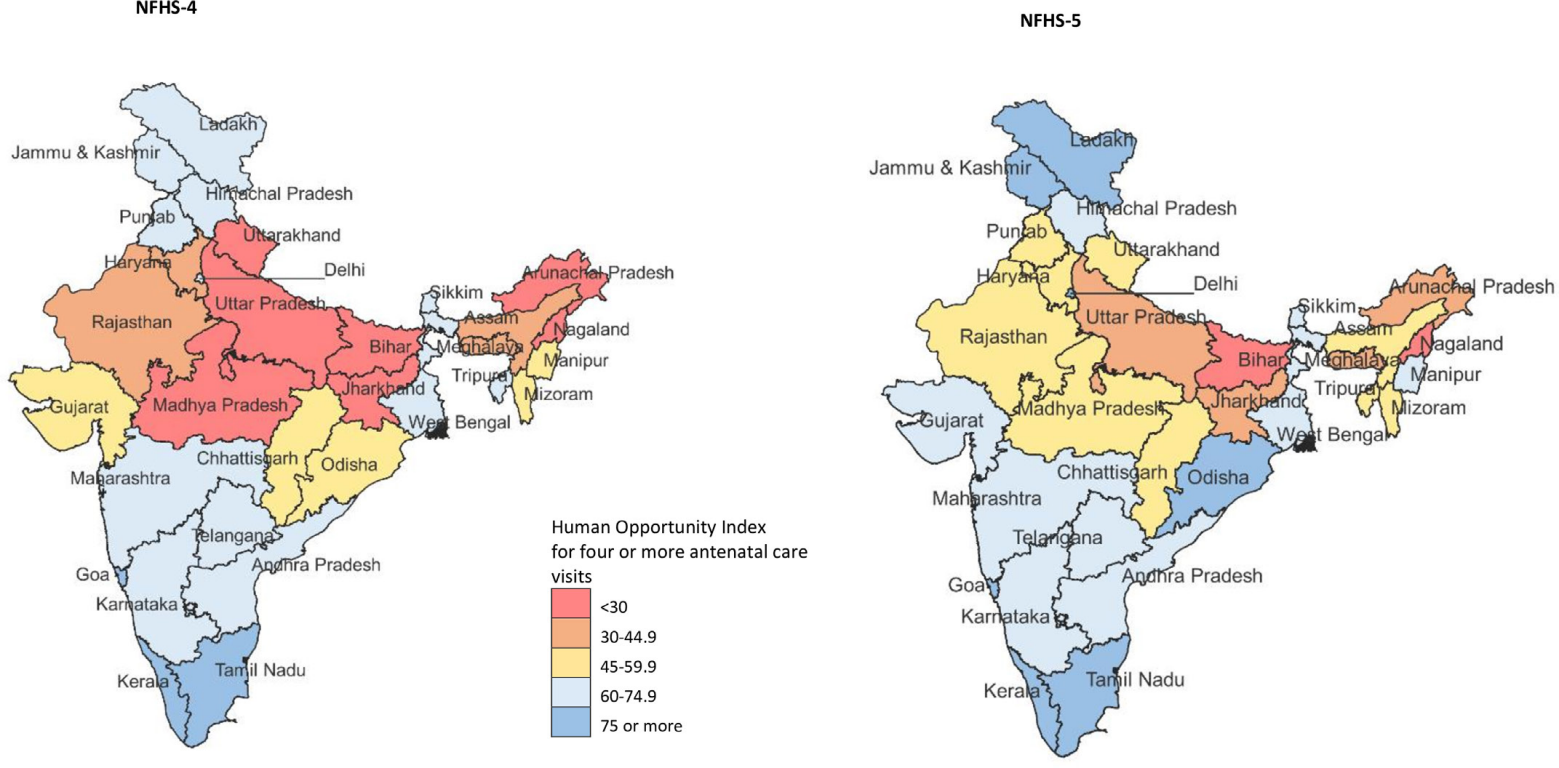
Human Opportunity Index for 4+antenatal care visits in NFHS-4 in 2015–2016 and NFHS-5 in 2019–2021 for each state of India. NFHS, National Family Health Survey.

The coverage of 4+ANC visits for India was 57.0% (95% CI 56.8% to 57.3%) in NFHS-5. The per cent change in coverage of 4+ANC visits from NFHS-4 to NFHS-5 was estimated at 22.8% (95% CI 22.1% to 23.5%) for India, 33.3% (95% CI 32.3% to 34.3%) for less developed and 3.4% (95% CI 2.6% to 4.1%) for more developed states (online supplemental table 2). This coverage ranged from 15.6% in Nagaland to 80.0% in Odisha in the less developed states, and 58.3% in Punjab to 93.2% in Goa in the more developed states in NFHS-5 (online supplemental table 2). The HOI for geographical inequality in 4+ANC visits for India was 48.4 in NFHS-5, an improvement of 35.2% (95% CI 34.5% to 35.9%) between NFHS-4 to NFHS-5 (online supplemental table 2) and was substantially higher for the more developed states at 68.2 than the less developed states at 40.7 in NFHS-5 (table 1 and online supplemental table 3). HOI ranged from 8.3 to 88.9 in the states in NFHS-4, and 11.3 to 92.4 in the states in NFHS-5 (figure 1 and online supplemental table 3). Three and six states from the less and more developed states showed a reduction in HOI between NFHS-4 to NFHS-5, respectively (online supplemental table 3). The highest gains in HOI were made in the state of Uttarakhand (115.1%; 95% CI 109.0% to 121.1%) between NFHS-4 to NFHS-5, with the HOI for the state at 55.7 in NHFS-5.

**Table 1:** Overall Human Opportunity Index (HOI) and by wealth index quintile (WI) for four or more antenatal care visits and the ratio between WI I and overall HOI, WI V and overall HOI for India and its states, National Family Health Survey-5

	Overall HOI	Four or more ANC visits						
		HOI by WI					HOI ratio	
		I	II	III	IV	V	WI I: Overall HOI	WI V: Overall HOI
**India**	48.4	33.8	44.0	53.1	59.3	64.4	0.70	1.33
Less developed states	40.7	31.1	38.9	45.4	50.1	57.0	0.76	1.40
Arunachal Pradesh	34.3	20.2	31.6	37.0	44.2	47.3	0.59	1.38
Assam	45.6	38.9	46.4	53.3	60.1	61.5	0.85	1.35
Bihar	22.9	15.6	22.9	30.2	37.4	49.2	0.68	2.15
Chhattisgarh	57.2	54.3	53.4	60.9	60.5	61.0	0.95	1.07
Jharkhand	36.0	28.6	38.5	41.9	51.8	56.0	0.79	1.56
Madhya Pradesh	52.2	44.6	49.3	56.1	59.1	62.3	0.85	1.19
Manipur	61.2	36.7	64.6	78.7	84.9	91.3	0.60	1.49
Meghalaya	45.4	36.8	45.8	50.0	61.5	55.5	0.81	1.22
Mizoram	46.6	11.0	37.8	49.9	61.5	69.9	0.24	1.50
Nagaland	11.3	5.4	10.1	20.8	26.9	20.0	0.48	1.77
Odisha	76.2	69.3	78.6	79.1	81.6	82.4	0.91	1.08
Rajasthan	51.2	45.7	45.9	49.6	52.2	59.0	0.89	1.15
Sikkim	60.1	66.9	61.1	59.1	61.3	47.6	1.11	0.79
Tripura	50.4	41.7	52.8	56.6	64.6	51.2	0.83	1.02
Uttar Pradesh	38.1	30.4	35.0	38.9	41.4	50.9	0.80	1.34
Uttarakhand	55.7	35.2	44.2	51.8	62.6	69.1	0.63	1.24
More developed states	68.2	58.6	65.1	67.1	70.1	70.5	0.86	1.03
Andhra Pradesh	68.3	45.5	64.2	64.8	66.5	69.2	0.67	1.01
Goa	92.4	100.0	81.2	89.8	92.5	93.5	1.08	1.01
Gujarat	73.1	63.2	69.1	72.4	75.9	80.5	0.86	1.10
Haryana	56.6	33.5	44.8	49.7	53.3	64.5	0.59	1.14
Himachal Pradesh	67.1	52.1	53.4	62.3	74.2	74.8	0.78	1.11
Jammu and Kashmir	76.3	60.2	75.8	76.7	80.6	79.5	0.79	1.04
Karnataka	65.1	51.8	60.3	65.4	71.2	69.5	0.80	1.07
Kerala	77.3	71.3	87.6	76.0	78.6	74.8	0.92	0.97
Maharashtra	66.7	49.9	63.3	65.1	71.2	73.8	0.75	1.11
Delhi	75.3	26.6	45.1	62.2	68.6	78.8	0.35	1.05
Punjab	54.7	31.2	41.6	47.1	50.7	57.6	0.57	1.05
Tamil Nadu	89.6	88.3	89.1	89.1	90.3	87	0.99	0.97
Telangana	68.2	53.5	63.9	64.8	70	68.5	0.78	1.00
West Bengal	72.2	66.5	72.1	75.0	78.7	78	0.92	1.08

Ratio of HOI: 
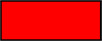
, Lless than 0.50 ; 
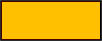
, 0.51–0.89 ; 
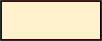
, 0.90–1.09 ; 
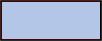
, 1.10–1.49 ; 
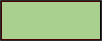
, 1.50 or more.

ANCantenatal care

Considering WI (table 1), HOI ranged from 33.8 for WI I to 64.4 for WI V for India, and the corresponding range was 31.1–57.0 and 58.6–70.5 for less and more developed states in NFHS-5, respectively. The HOI ratio of WI V to average was 1 or more in all states except Sikkim, Tamil Nadu and Kerala. Substantial variations were seen in HOI ratio of WI I–V, ranging from 0.16 to 1.41, with this ratio being <1 in 27 (90.0%) states in NFHS-5, and the lowest HOI ratio between WI I and V was seen in Mizoram at 0.16 (online supplemental table 4). Among the 20 states with WI I to state HOI ratio of >0.70, 15 states had the W1 I–WI V HOI ratio of <0.70 (online supplemental table 4).

### Institutional delivery with SBA

**Figure 2:**
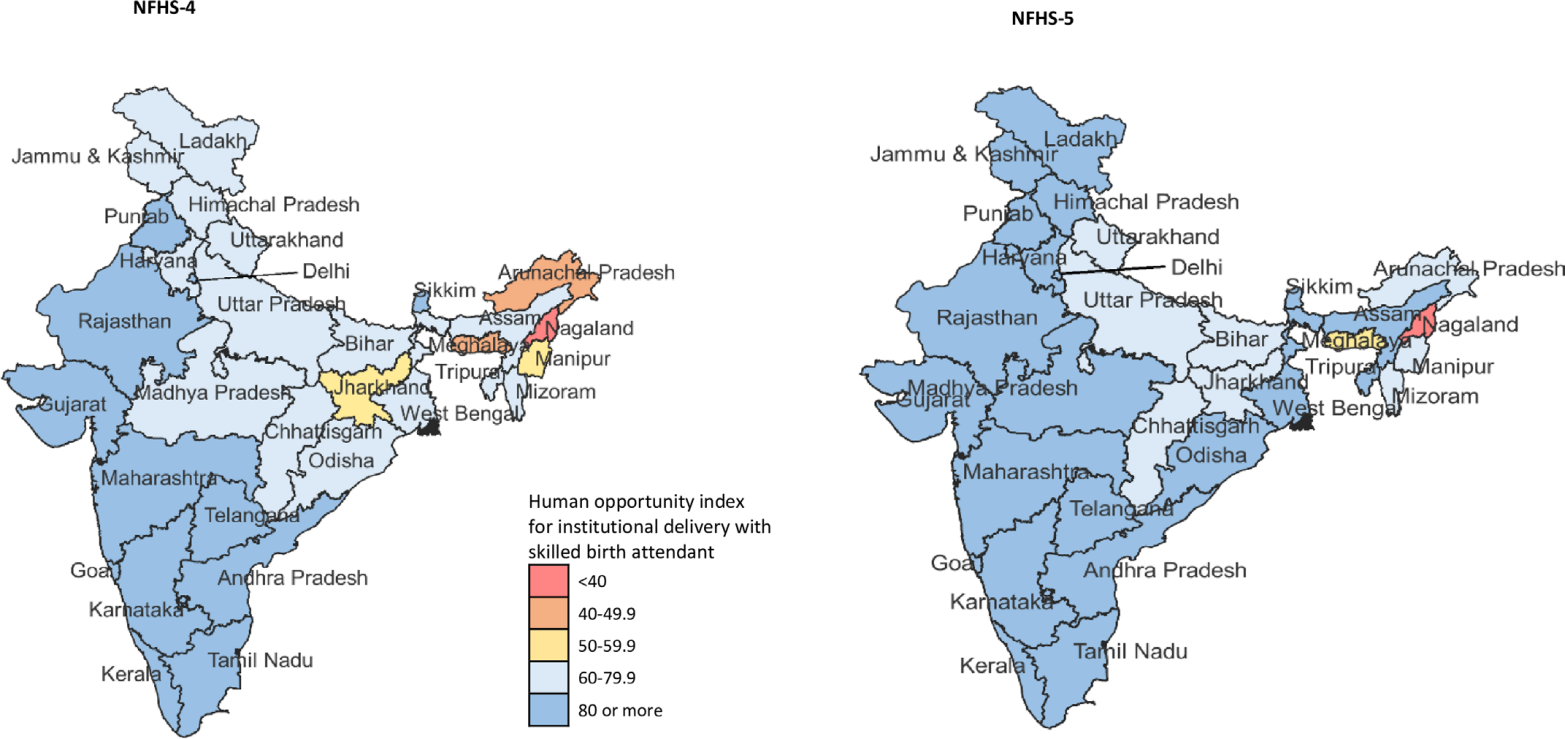
Human Opportunity Index for institutional delivery with skilled birth attendant in NFHS-4 in 2015–2016 and NFHS-5 in 2019–202021 for each state of India. NFHS, National Family Health Survey.

The coverage of institutional delivery with SBA for India was 85.0% (95% CI 84.9% to 85.2%) in NFHS-5. The per cent change from NFHS-4 to NFHS-5 was estimated at 12.6% (95% CI 12.2% to 12.9%) for India, 14.6% (95% CI 14.1% to 15.1%) for less developed and 6.2% (95% CI 5.8% to 6.6%) for more developed states (online supplemental table 2). This coverage ranged from 58.6% in Meghalaya to 96.3% in Sikkim in less developed states and 84.3% in Himachal Pradesh to 99.8% in Kerala in more developed states in NFHS-5 (online supplemental table 2). The HOI for geographical inequality for institutional delivery with SBA for India was 80.4, 90.1 and 76.2 for India, more developed and less developed states in NFHS-5, respectively (table 2 and online supplemental table 3). HOI ranged from 26.2 to 99.8 in the states in NFHS-4, and 37.4 to 99.7 in the states in NFHS-5 (figure 2 and online supplemental table 3). The highest gains in HOI between NHFS-4 and NFHS-5 were made in Arunachal Pradesh state (64.9%; 95% CI 61.3% to 68.5%), with the HOI for the state at 77.5 in NHFS-5 (online supplemental table 3).

**Table 2:** Overall Human Opportunity Index (HOI) by wealth index quartile (WI) for institutional delivery with skilled birth attendant (SBA) and the ratio between WI I and overall HOI, WI V and overall HOI for India and its states, National Family Health Survey-5

	Overall HOI	Institutional delivery with SBA						
		HOI by WI					HOI ratio	
		I	II	III	IV	V	WI I: Overall HOI	WI V: Overall HOI
**India**	80.4	65.1	78.8	86.0	90.2	93.7	0.81	1.17
Less developed states	76.2	63.6	76.6	83.5	87.8	92.0	0.83	1.21
Arunachal Pradesh	77.5	56.0	77.6	86.7	92.0	96.0	0.72	1.24
Assam	82.3	72.1	87.0	91.7	94.8	95.5	0.88	1.16
Bihar	72.1	63.9	76.6	81.2	82.9	88.7	0.89	1.23
Chhattisgarh	78.5	68.0	80.1	85.9	90.3	94.3	0.87	1.20
Jharkhand	70.4	60.7	77.1	83.8	91.3	94.7	0.86	1.35
Madhya Pradesh	84.1	75.4	84.8	90.0	91.7	90.3	0.90	1.07
Manipur	62.0	37.7	65.1	78.7	88.2	93.7	0.61	1.51
Meghalaya	52.5	38.8	57.6	72.6	82.5	82.2	0.74	1.57
Mizoram	72.4	26.4	59.1	80.5	92.8	95.8	0.36	1.32
Nagaland	37.4	26.2	37.9	57.1	64.7	74.9	0.70	2.00
Odisha	87.3	77.6	91.6	93.9	95.5	95.6	0.89	1.10
Rajasthan	93.9	89.2	91.8	94.1	94.8	97.1	0.95	1.03
Sikkim	95.2	88.5	94.5	94.8	97.3	94.2	0.93	0.99
Tripura	83.5	71.8	88.8	91.9	94.0	90.6	0.86	1.09
Uttar Pradesh	78.0	68.3	76.4	79.4	82.8	90.1	0.88	1.16
Uttarakhand	78.5	62.1	62.7	75.0	85.7	91.9	0.79	1.17
More developed states	90.1	76.5	85.8	90.1	92.6	95.1	0.85	1.06
Andhra Pradesh	95.4	73.9	93.0	94.2	95.9	98.7	0.77	1.03
Goa	98.5	100.0	90.6	100.0	98.5	98.7	1.02	1.00
Gujarat	87.5	75.3	85.9	88.8	92.1	91.1	0.86	1.04
Haryana	90.5	64.7	77.0	86.8	91.1	96.0	0.71	1.06
Himachal Pradesh	82.5	54.1	68.1	80.8	86.5	90.6	0.66	1.10
Jammu and Kashmir	87.9	71.5	82.7	88.6	94.7	96.6	0.81	1.10
Karnataka	90.3	83.8	85.6	90.6	93.8	93.2	0.93	1.03
Kerala	99.7	100.0	100.0	99.5	99.8	99.6	1.00	1.00
Maharashtra	88.2	69.5	85.4	89.0	92.3	95.0	0.79	1.08
Delhi	90.2	21.9	68.7	82.7	83.0	91.6	0.24	1.02
Punjab	92.9	79.4	80.7	96.5	90.5	94.6	0.85	1.02
Tamil Nadu	99.2	95.6	97.9	99.2	99.5	100.0	0.96	1.01
Telangana	90.4	75.5	88.9	90.0	90.4	91.4	0.84	1.01
West Bengal	89.2	83.7	90.6	93.8	94.6	93.1	0.94	1.04

Ratio of HOI: 
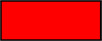
, Lless than 0.50; 
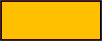
, 0.51–0.89; 
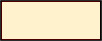
, 0.90–1.09; 
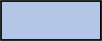
, 1.10–1.49; 
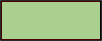
,1.50 or more.

Considering WI (table 2), HOI ranged from 65.1 for WI I to 93.7 for WI V for India, and the corresponding range was 63.6–92.0 and 76.5–95.1 for less and more developed states in NFHS-5, respectively. The WI V to average HOI ratio was 1 or more in all the states except Sikkim. The HOI ratio of WI I to WI V was <1 in 28 (93.3 %) states and was <0.60 in 6 (20.0 %) states in NFHS-5 (online supplemental table 4). Among the 26 states with W1 1 to state HOI ratio being >0.70, 6 states had the W1 I to WI V HOI ratio of <0.70 (online supplemental table 4).

### PNC within 48 hours of delivery

**Figure 3:**
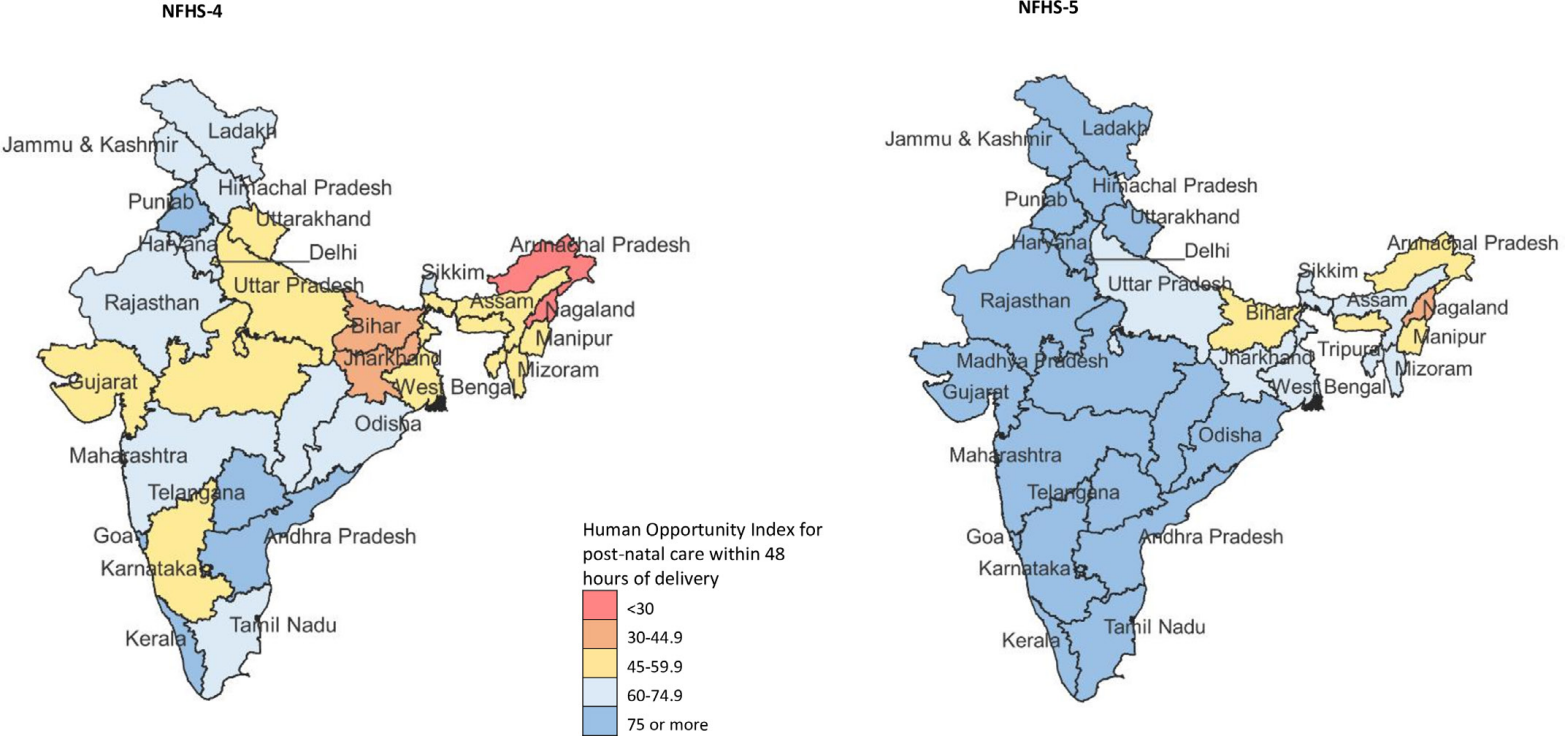
Human Opportunity Index for postnatal care within 48 hours of delivery in NFHS-4 in 2015–2016 and NFHS-5 in 2019–2021 for each state of India. NFHS, National Family Health Survey.

The coverage of PNC within 48 hours of delivery was 78.9% (95% CI 78.7% to 79.1%) in NFHS-5. The per cent change from NFHS-4 to NFHS-5 was estimated at 28.5% (95% CI 28.0% to 29.0%) for India, 31.8% (95 % CI 31.1% to 32.4%) for less developed and 19.6% (95% CI 19.0% to 20.3%) for more developed states (online supplemental table 2). This coverage ranged from 43.2% in Nagaland to 92.1% in Odisha in the less developed states and 68.2% in West Bengal to 96.3% in Goa in the more developed states in NFHS-5 (online supplemental table 2). The HOI for geographical inequality in PNC within 48 hours of delivery for India was 73.5 in NFHS-5, which improved by 34.6% (95% CI 34.2% to 35.1%) between NFHS-4 to NFHS-5. The HOI was substantially higher for the more developed states 82.6 than the less developed states 69.4 in NFHS-5 (table 3 and online supplemental table 3). HOI ranged from 17.2 to 90.5 in the states in NFHS-4, and 37.5 to 95.6 in the states in NFHS-5 (figure 3 and online supplemental table 3). The highest gains in HOI were made in Arunachal Pradesh (128.6%; 95% CI 122.1% to 135.2%) and Nagaland (118.0%; 95% CI 108.5% to 127.5%), with the HOI for the states at 51.9 and 37.5 in NHFS-5, respectively (online supplemental table 3).

**Table 3:** Overall Human opportunity index (HOI) by wealth index quartile (WI) for postnatal care within 48 hours of delivery and the ratio between WI I and overall HOI, WI V and overall HOI for India and its states, National Family Health Survey-5

	Overall HOI	PNC within 48 hours of delivery						
		HOI by WI					HOI ratio	
		I	II	III	IV	V	WI I: Overall HOI	WI V: Overall HOI
**India**	73.5	62.0	70.7	77.1	81.3	85.0	0.84	1.16
Less developed states	69.4	61.2	68.7	73.6	77.4	83.0	0.88	1.20
Arunachal Pradesh	51.9	41.5	52.6	53.2	53.6	59.6	0.80	1.15
Assam	64.7	56.8	67.4	72.7	74.4	72.0	0.88	1.11
Bihar	59.6	54.0	61.8	65.8	69.0	75.8	0.91	1.27
Chhattisgarh	85.3	81.8	85.5	85.2	89.4	87.8	0.96	1.03
Jharkhand	70.9	65.8	74.6	74.5	81.4	81.4	0.93	1.15
Madhya Pradesh	82.8	78.7	81.5	84.4	85.7	86.7	0.95	1.05
Manipur	57.7	38.1	59.2	72.7	78.1	79.0	0.66	1.37
Meghalaya	53.4	49.3	55.6	59.3	54.9	56.3	0.92	1.05
Mizoram	63.9	28.2	55.0	72.2	72.1	77.3	0.44	1.21
Nagaland	37.5	27.2	38.8	55.2	59.8	57.5	0.73	1.53
Odisha	90.7	87.6	90.7	92.9	92.9	92.9	0.97	1.02
Rajasthan	82.6	78.2	80.8	82.3	83.4	85.2	0.95	1.03
Sikkim	70.4	66.9	69.3	71.4	70.6	57.4	0.95	0.82
Tripura	64.2	56.4	63.0	72.9	79.1	82.8	0.88	1.29
Uttar Pradesh	73.2	66.0	72.6	73.6	77.3	82.8	0.90	1.13
Uttarakhand	81.5	71.1	73.6	80.9	85.4	87.3	0.87	1.07
More developed states	82.6	67.4	77.2	83.0	85.3	88.3	0.82	1.07
Andhra Pradesh	90.4	67.6	87.2	91.1	90.3	92.1	0.75	1.02
Goa	95.6	100.0	90.6	89.8	96.6	98.7	1.05	1.03
Gujarat	87.1	85.7	85.4	85.8	86.3	89.3	0.98	1.03
Haryana	88.7	64.2	76.7	88.7	88.7	92.2	0.72	1.04
Himachal Pradesh	87.3	71.3	78.7	88.3	88.3	92.1	0.82	1.05
Jammu and Kashmir	77.2	64.7	72.9	78.1	80.3	82.5	0.84	1.07
Karnataka	83.6	76.3	80.7	84.7	85.5	83.8	0.91	1.00
Kerala	91.5	81.6	83.9	88.3	92.1	91.7	0.89	1.00
Maharashtra	80.8	66.9	78.3	80.7	83.3	86.0	0.83	1.06
Delhi	85.1	31.2	59.7	76.4	80.3	87.2	0.37	1.02
Punjab	85.4	69.4	75.0	96.6	83.1	86.6	0.81	1.01
Tamil Nadu	90.4	84.0	90.4	90.3	90.1	89.4	0.93	0.99
Telangana	85.4	78.1	83.1	83.5	84.4	88.2	0.91	1.03
West Bengal	63.8	56.2	62.0	67.6	76.4	80.4	0.88	1.26

Ratio of HOI: 
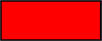
, Lless than 0.50; 
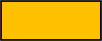
, 0.51–0.89; 
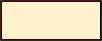
, 0.90–1.09; 
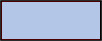
, 1.10–1.49; 
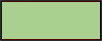
, 1.50 or more.

PNCpostnatal care

Considering WI (table 3), HOI ranged from 62.0 for WI I to 85.0 for WI V in India, and the corresponding range was from 61.2 to 83.0 and 67.4 to 88.3 for less and more developed states in NFHS-5, respectively. The HOI ratio of WI V to state was 1 or more in all the states, except for Sikkim and Tamil Nadu. The HOI ratio of WI I to WI V was <1 in 28 (93.3%) states and was <0.60 in 4 (13.3%) states in NFHS-5 (online supplemental table 4). Among the 27 states with WI I to state HOI ratio of >0.70, 2 states had the WI I to WI V HOI ratio of <0.70 (online supplemental table 4).

### Continuum of care

**Figure 4:**
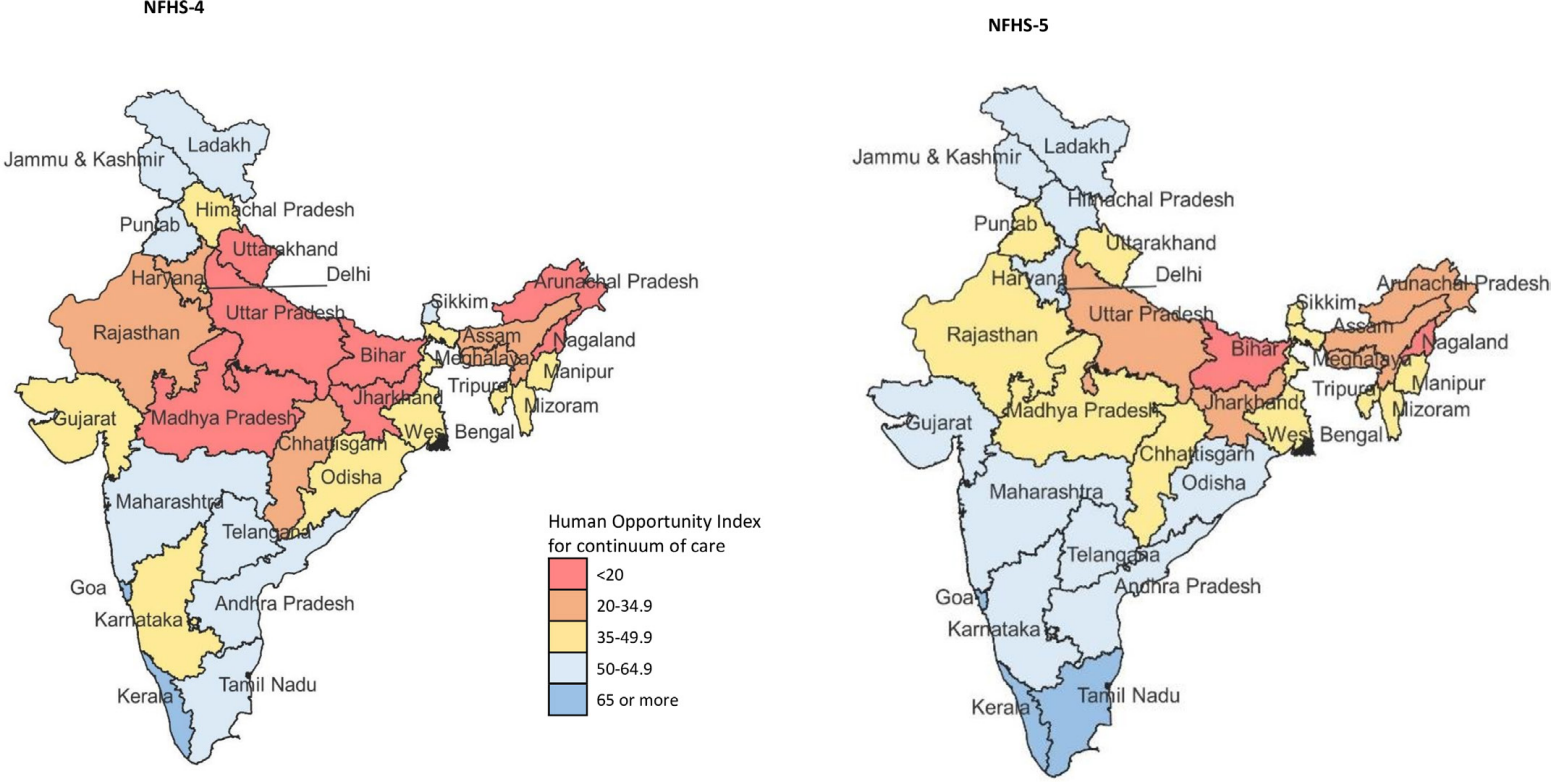
Human Opportunity Index for continuum of care in NFHS-4 in 2015–2016 and NFHS-5 in 2019–2021 for each state of India. NFHS, National Family Health Survey.

The coverage of CoC was 45.6% (95% CI 45.4% to 45.9%) in NFHS-5. The per cent change from NFHS-4 to NFHS-5 was estimated at 38.6% (95% CI 37.6% to 39.6%) for India, 47.2% (95% CI 45.7% to 48.6%) for less developed and 19.5% (95% CI 18.4% to 20.6%) for more developed states (online supplemental table 2). The coverage ranged from 9.4% in Nagaland to 69.4% in Odisha in the less developed states, and 50.2% in Punjab to 90.1% in Goa in the more developed states in NFHS-5 (online supplemental table 2). The HOI for geographical inequality in CoC for India was 37.1, which improved by 52.2% (95% CI 51.1% to 53.0%) between NFHS-4 to NFHS-5. The HOI was substantially higher for the more developed states at 56.3 than the less developed states at 29.7 in NFHS-5 (table 4 and online supplemental table 3). HOI ranged from 4.4 to 79.0 in the states in NFHS-4, and 6.5 to 88.8 in the states in NFHS-5 (figure 4 and online supplemental table 3). The highest gains in HOI were made in Arunachal Pradesh (167.1%; 95% CI 154.4% to 179.8%) and Uttarakhand (147.4%; 95% CI 139.3% to 155.5%), with the HOI for the states at 21.9 and 43.3 in NFHS-5, respectively (online supplemental table 3).

**Table 4:** Overall Human Opportunity Index (HOI) by wealth index quartile (WI) for continuum of care and the ratio between WI I and overall HOI, WI V and overall HOI based on the maternal services for India and its states, National Family Health Survey-5

	Overall HOI	Continuum of care						
		HOI by WI					HOI ratio	
		I	II	III	IV	V	WI I: Overall HOI	WI V: Overall HOI
**India**	37.1	21.7	32.3	41.9	49.2	56.5	0.58	1.52
Less developed states	29.7	19.8	27.9	34.5	40.2	48.8	0.67	1.64
Arunachal Pradesh	21.9	12.4	20.9	22.8	26.0	32.8	0.57	1.50
Assam	31.0	22.9	32.1	40.3	49.0	44.3	0.74	1.43
Bihar	15.3	9.5	15.1	21.0	28.4	38.6	0.62	2.52
Chhattisgarh	43.3	37.3	39.2	47.9	50.7	53.4	0.86	1.23
Jharkhand	24.1	16.7	27.8	31.6	41.5	45.9	0.69	1.90
Madhya Pradesh	41.2	31.7	39.0	46.5	50.7	53.2	0.77	1.29
Manipur	41.5	19.8	41.3	60.2	68.0	67.1	0.48	1.62
Meghalaya	20.5	14.1	21.7	27.6	34.7	28.1	0.69	1.37
Mizoram	36.6	6.7	27.6	40.4	49.5	57.3	0.18	1.57
Nagaland	6.5	2.2	5.9	12.2	17.4	35.6	0.34	5.48
Odisha	64.8	53.2	68.2	71.9	75.1	77.3	0.82	1.19
Rajasthan	44.2	38.0	39.8	42.3	45.2	52.0	0.86	1.18
Sikkim	48.3	47.6	49.0	45.6	50.3	37.6	0.99	0.78
Tripura	35.3	19.5	37.4	39.8	56.2	51.2	0.55	1.45
Uttar Pradesh	27.9	19.2	24.9	28.5	31.7	43.7	0.69	1.57
Uttarakhand	43.3	24.5	28.2	38.5	51.2	59.2	0.57	1.37
More developed states	56.3	39.3	50.0	55.5	59.8	62.7	0.70	1.11
Andhra Pradesh	59.3	32.4	53.6	59.6	60.5	64.9	0.55	1.09
Goa	88.8	100.0	71.9	86.4	87.2	91.6	1.13	1.03
Gujarat	61.2	49.8	56.9	60.0	64.0	69.9	0.81	1.14
Haryana	51.2	23.3	35.5	45.0	47.2	60.3	0.46	1.18
Himachal Pradesh	54.3	31.8	39.5	51.0	59.7	65.1	0.59	1.20
Jammu and Kashmir	59.5	40.2	53.9	62.3	66.1	66.0	0.68	1.11
Karnataka	52.6	36.3	47.1	53.9	59.9	56.4	0.69	1.07
Kerala	72.9	68.0	69.1	70.1	74.5	71.6	0.93	0.98
Maharashtra	54.5	33.5	49.9	51.8	60.3	65.9	0.61	1.21
Delhi	65.3	1.6	28.0	47.9	55.4	68.8	0.02	1.05
Punjab	46.9	28.7	31.5	95.8	41.8	50.1	0.61	1.07
Tamil Nadu	82.0	75.6	81.9	81.7	82.6	79.1	0.92	0.96
Telangana	54.9	35.9	51.0	52.2	56.2	57.0	0.65	1.04
West Bengal	47.3	37.7	45.6	52.3	61.3	65.6	0.80	1.39

Ratio of HOI: 
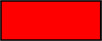
, Lless than 0.50; 
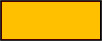
, 0.51–0.89; 
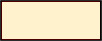
, 0.90–1.09; 
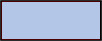
, 1.10–1.49; 
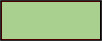
, 1.50 or more.

Considering WI (table 4), HOI ranged from 21.7 for WI I to 56.5 for WI V for India, and the corresponding range was 19.8 to 48.8 and 39.3 to 62.7 for less and more developed states in NFHS-5, respectively. The HOI ratio of WI V to state was 1 or more in 27 states. The HOI ratio of WI I to WI V was <1 in 28 (93.3%) states and was <0.60 in 18 (60.0%) states in NFHS-5 (online supplemental table 4). Among the 12 states with WI I to state HOI ratio of >0.70, 3 states the WI I to WI V HOI ratio of <0.70 (online supplemental table 4).

## Discussion

This analysis of coverage of maternal health services for live births over a decade between NFHS-4 and NFHS-5 has highlighted significant improvements in the geographical inequity for coverage of these services at the state level in India and has identified the inequities that remain to be addressed to achieve the ENAP 2025 coverage targets for these services. The geographical inequity for the coverage of 4+ANC visits lags significantly behind that of institutional delivery with SBA and PNC within 48 hours of delivery, contributing to the CoC inequity in NFHS-5. The HOI ratio for CoC at 0.38 highlights the extent of inequity between the women belonging to the lowest and the highest WI quintiles in India.

A policy aiming at equitable access would require progress towards two objectives: first, expanding the coverage by ensuring that as many women as possible get the opportunity; and second, by allocating new opportunities first to the vulnerable population who are at a disadvantage due to their circumstances.^29^ We used HOI,^25^ as a tool to measure the distribution of opportunities and equitable access to maternal health services. The HOI not only carries information about the coverage rate of the service but also how fairly the available services are distributed among women of different backgrounds, in this case of different WI quintiles.^30^ In terms of expanding coverage to ensure that as many women as possible get the opportunity, the coverage of 4+ANC visits was for four of seven women and that for institutional delivery with SBA was six out of seven women, and for PNC within 48 hours of delivery was four out of five women in NFHS-5. The differential increase over the decade between the coverages of these three maternal health services can possibly be explained by how the national programmes to reduce maternal and neonatal mortality were implemented during this period. The programme to address ANC services was launched in 2016 after NFHS-4 and is aimed to guarantee a minimum package of ANC services to women in their second/third trimesters of pregnancy at designated government health facilities.^31^ There is no monetary incentive either to the health worker or to the pregnant women under this programme. The likely impact of this programme on HOI for 4+ANC visits coverage will be documented in the next round of NFHS (round 6). However, given the extent of low coverage of 4+ANC visits in most of the less developed states and some of the more developed states, major inputs are needed to address the barriers for improved ANC utilisation to achieve the ENAP 2025 target for 4+ANC visits.^32^,^34^ The increased coverage for institutional deliveries with SBA between the two surveys resulted in nearly doubling of HOI over this period, and near universality of this service in the more developed states. Two programmes—the Janani Suraksha Yojana (JSY) and the Janani Shishu Suraksha Karyakaram (JSSK)—initiated to increase the coverage of institutional deliveries in India have contributed to the exponential increase in access to institutional delivery.^35 36^ The JSY was initiated in 2005 and was a large-scale national programme that offered conditional cash transfer and support services to poor pregnant women to use institutional delivery care facilities, especially in the states with lower coverage.^35^ The JSSK, initiated in 2011, provided free and cashless delivery along with some other benefits to pregnant women to eliminate out-of-pocket expenses in order to increase the institutional deliveries.^37^ The PNC coverage was estimated at 61% HOI at 73.5 for India in NFHS-5 with a wide variation at the state level. The increased PNC coverage also resulted in 34.6% increase in HOI for PNC coverage between NFHS-4 and NFHS-5. According to the Indian PNC guidelines,^38^ the health workers are paid INR250 (US$3.5) for PNC visits and are expected to undertake 6–7 PNC visits at home from 1st to 42nd day of delivery for counselling the mother on various issues and enabling referral if needed. Considering all the three maternal health services together, the CoC coverage remained at 62.5% in NFHS-5 with only 19.5% change between the two surveys even in the more developed states. Only 45% CoC coverage in NFHS-5 for India translates into 5 out of every 11 women and newborns not having received all the three MNCH services, which is the basic premise of the ENAP and INAP to address neonatal and maternal mortality.^19 21^

With regard to the policy objective of allocating new opportunities to the vulnerable population who are at a disadvantage due to their circumstances, socioeconomic inequities in maternal health services in India have been reported previously,^1639^,^45^ but the use of HOI to measure inequity has not been attempted. For all the maternal health services, the women belonging to the lowest WI quintile had low HOI compared with those in the highest WI quintile, and the difference was starker for ANC services and in the less developed states. Though the within-district inequalities in intervention coverages are reducing in most states, the pace of reduction has not been the same for every woman. The state of Delhi, the national capital, had the one of the least HOI ratio for the women belonging to the lowest WI quintile. The extent of HOI ratio for the lowest to the highest WI quintile and that to average HOI between the maternal health services within states highlights the specificity needed in targeting women with the respective interventions to achieve 2025 coverage targets. One of the challenges in achieving equity in maternal health services in India is the dependence on NFHS for retrospective situational analysis rather than for monitoring and evaluation because the routine health information management system(HMIS) does not allow for tracking of CoC for maternal health services per woman.^46^ Real-time tracking of women for utilisation of these services at the district level is needed to reduce inequity in the coverage of these services. India has a Mother and Child Tracking System (MCTS) in place since 2009 to follow a mother–child dyad through the CoC; however, it is known to be fraught with several issues.^47^,^49^ Clearly, it is important that strategies to improve the MCTS, within digital health, are looked into urgently if India aims to to reach every woman and newborn to achieve the SDG targets of reduced maternal and neonatal mortality at the district level.^50^,^52^ High-quality timely data for evidence-informed decision-making to reduce inequity at the district level can also be obtained through continuous surveys.^53^ Furthermore, incentivisation of services is currently only for institutional delivery and for PNC services. With poor coverage of ANC services resulting in poor coverage of CoC, it may be worth considering incentivising the CoC rather than individual maternal health services to address inequity.

The major strength of this analysis is the utilisation of HOI to indicate not only inequity in the coverage rate of the services but also how fairly the available services are distributed among women by geography and WI quintiles. This, we believe, allows for understanding the women who need to be targeted to reduce the inequity in the maternal healthcare in India. The inclusion of CoC is another strength as it brings the woman to the centre stage as against the individual maternal healthcare services. The results from this analysis are generalisable as the sample for the NFHS is nationally representative of ever-married women who reported a livebirth in the last 5 years from the time of data collection. Hence, owing to the large sample size and broad geographical coverage strengthens the external validity of these findings ensuring applicability across different population in India.

Some limitations of the analysis presented should be taken into account. The data on service utilisation documented in the NFHS are self-reported by women, which may be subject to recall bias. We believe this bias to be of less concern as women self-report accurately indicators related to concrete and observable actions performed on them as opposed to information or advice they were offered.^54 55^ We only considered the inequity in coverage and not in the quality of contact with the health system during service utilisation in this analysis as such data are not available in the NFHS. There is a growing literature documenting increased coverage but poor quality of contact for these services,^4^ including from India from pregnancy to delivery,^449 56^,^66^ and also of poor quality of healthcare is a major driver of excess mortality across conditions, including neonatal mortality.^67^,^70^ Health system redesign has been suggested for equity in maternal and newborn health, by moving all childbirth care services to hospitals in all countries, combined with improvements in the quality of care provided in these facilities, transportation from home to hospital and continuity of care through hub-and-spoke arrangements.^71^ While commenting on the scope of health system redesign is beyond the scope of the analysis undertaken for this paper, more discussion is needed and more options need to be rigorously tried and tested to develop sustainable district health systems which are fit for purpose and respond to continuity of care needs of women and their babies.^72^

## Conclusion

In conclusion, the findings of this assessment of coverage of maternal health services for live births over a decade are encouraging as significant reductions in geographical inequity are documented. However, the findings also emphasise the need for improved targeting of women to reduce the remaining inequity gap to achieve the ENAP targets, in particular for the coverage of 4+ANC visits. It will be important for the national programme to monitor the CoC coverage in real time in addition to the three maternal health services individually to monitor and track inequity at the district level for every pregnant woman to ensure that inequity by wealth index is also addressed in India.

## supplementary material

10.1136/bmjopen-2024-083922 online supplemental file 1

## Data Availability

All data relevant to the study are included in the article or uploaded as online supplemental information.
