# Supplementary material for: Population-level trends over a decade in geographical inequality for opportunity in access to maternal care services: a cross-sectional analysis from the National Family Health Surveys in India
Source: BMJ Open. 2024 Nov 21;14(11):e083922. doi: 10.1136/bmjopen-2024-083922 (PMC11590786; doi:10.1136/bmjopen-2024-083922)
Supplement: online supplemental file 1 [file bmjopen-14-11-s001.pdf]

Population level trends over a decade in geographical inequality for opportunity in access to maternal care services: findings from the National Family Health Surveys in India

Supplementary file

Correspondence to: Prof. Rakhi Dandona; rakhi.dandona@phfi.org

Supplementary Table 1. Number of districts by state in the National Family Health Survey (NFHS) rounds 4 and 5.

	Number of districts	
	**NFHS-4**	**NFHS-5**
**India**	**628**	**695**
**Less developed**	**363**	**395**
Arunachal Pradesh	16	20
Assam	27	33
Bihar	38	38
Chhattisgarh	18	27
Jharkhand	24	24
Madhya Pradesh	50	51
Manipur	9	9
Meghalaya	7	11
Mizoram	8	8
Nagaland	11	11
Odisha	30	30
Rajasthan	33	33
Sikkim	4	4
Tripura	4	8
Uttar Pradesh	71	75
Uttarakhand	13	13
**More developed**	**265**	**300**
Andhra Pradesh	13	13
Delhi	9	11
Goa	2	2
Gujarat	26	33
Haryana	21	22
Himachal Pradesh	12	12
Jammu & Kashmir[§]	22	22
Karnataka	30	30
Kerala	14	14
Maharashtra	35	36
Punjab	20	22
Tamil Nadu	32	32
Telangana	10	31
West Bengal	19	20

Supplementary Table 2. Coverage of four of more antenatal care (ANC) visits, institutional delivery with skilled birth attendant (SBA), and post-natal care within 48 hours of delivery, and continuum of care for the most recent livebirth in the last 5 years in the National Family Health Survey (NFHS) 4 and NFHS 5 for India and its states. CI denotes confidence interval.

State	Sample size		Four of more ANC visits			Institutional delivery with SBA*			Post-natal care within 48 hours of delivery			Continuum of care		
	NFHS-4 (N)	NFHS-5 (N)	NFHS-4<br>% of N (95% CI)	NFHS-5<br>% of N (95% CI)	Percent change from NFHS-4 to NFHS-5 (95% CI)	NFHS-4<br>% of N (95% CI)	NFHS-5<br>% of N (95% CI)	Percent change from NFHS-4 to NFHS-5 (95% CI)	NFHS-4<br>% of N (95% CI)	NFHS-5<br>% of N (95% CI)	Percent change from NFHS-4 to NFHS-5 (95% CI)	NFHS-4<br>% of N (95% CI)	NFHS-5<br>% of N (95% CI)	Percent change from NFHS-4 to NFHS-5 (95% CI)
**India**	**188,506**[†]	**174,796**	**46.4<br>(46.2-46.7)**	**57.0<br>(56.8-57.3)**	**22.8<br>(22.1-23.5)**	**75.5<br>(75.3-75.7)**	**85.0<br>(84.9-85.2)**	**12.6<br>(12.2-12.9)**	**61.4<br>(61.2 - 61.6)**	**78.9<br>(78.7-79.1)**	**28.5<br>(28.0-29.0)**	**32.9<br>(32.7-33.1)**	**45.6<br>(45.4-45.9)**	**38.6<br>(37.6-39.6)**
**Less developed**	**132,809**	**113,519**	**36.0<br>(35.7-36.3)**	**48.0<br>(47.7-48.3)**	**33.3<br>(32.3-34.3)**	**70.6<br>(70.3-70.8)**	**80.9<br>(80.7-81.2)**	**14.6<br>(14.1-15.1)**	**57.0<br>(56.7-57.2)**	**75.1<br>(74.8-75.3)**	**31.8<br>(31.1-32.4)**	**24.8<br>(24.6- 25.1)**	**36.5<br>(36.3-36.8)**	**47.2<br>(45.7-48.6)**
Arunachal Pradesh	3,858	4,570	24.6<br>(23.3- 26.0)	37.2<br>(35.8-38.6)	51.2<br>(45.1-57.4)	52.7<br>(51.1-54.2)	80.6<br>(79.4-81.7)	52.9<br>(49.8-56.1)	27.5<br>(26.1-28.9)	55.4<br>(53.9-56.8)	101.5<br>(95.7-107.2)	11.8<br>(10.8- 12.8)	25.3<br>(24.1-26.6)	114.7<br>(104.4-124.4)
Assam	8,534	9,247	45.4<br>(44.3- 46.4)	50.9<br>(49.8-51.9)	12.1<br>(9.5-14.7)	72.5<br>(71.5-73.4)	84.8<br>(84.1-85.5)	17.0<br>(15.6-18.4)	56.9<br>(55.8-57.9)	67.8<br>(66.8-68.7)	19.1<br>(17.1-21.2)	29.9<br>(20.9-30.9)	35.5<br>(34.5-36.5)	18.7<br>(15.1-22.4)
Bihar	16,822	13,874	14.6<br>(14.1 -15.2)	25.5<br>(24.7-26.2)	74.7<br>(70.0-79.3)	66.0<br>(65.3-66.7)	75.1<br>(74.4-75.8)	13.8<br>(12.5-15.0)	47.8<br>(47.1-48.6)	65.4<br>(64.6-66.2)	36.8<br>(35.0-38.7)	9.0<br>(8.6- 9.4)	17.8<br>(17.2-18.4)	97.8<br>(91.5-104.1)
Chhattisgarh	6,805	6,526	56.9<br>(55.7 - 58.0)	61.2<br>(60.0-62.4)	7.6<br>(5.3-9.9)	71.8<br>(70.7-72.8)	82.1<br>(81.2-83.0)	14.3<br>(12.7-16.0)	67.2<br>(66.0-68.3)	87.2<br>(86.3-87.9)	29.8<br>(28.0-31.5)	37.6<br>(36.4-38.7)	47.3<br>(46.1-48.5)	25.8<br>(22.4-29.2)
Jharkhand	8,947	7,465	29.9<br>(29.0 - 30.9)	38.6<br>(37.5-39.7)	29.1<br>(25.4-32.8)	63.8<br>(62.8-64.8)	74.5<br>(73.5-75.5)	16.8<br>(15.0-18.5)	46.8<br>(45.8-47.8)	74.0<br>(73.0-75.0)	58.1<br>(55.6-60.6)	17.0<br>(16.2-17.8)	27.0<br>(26.0-28.0)	58.8<br>(53.3-64.4)
Madhya Pradesh	17,406	11,700	34.9<br>(34.1-35.6)	57.0<br>(56.1-57.9)	63.3<br>(60.9-65.7)	75.9<br>(75.2-76.5)	86.6<br>(86.0-87.2)	14.1<br>(13.2-15.0)	55.9<br>(55.1-56.6)	85.1<br>(84.4-85.7)	52.2<br>(50.8-53.7)	23.1<br>(22.4 -23.7)	46.1<br>(45.2-47.0)	99.6<br>(96.2-102.9)
Manipur	4,429	2,511	65.1<br>(63.7-66.5)	68.7<br>(66.8-70.4)	5.5<br>(2.9-8.2)	67.9<br>(66.5-69.3)	70.9<br>(69.1-72.6)	4.4<br>(1.9-6.9)	62.8<br>(61.4-64.2)	64.9<br>(62.9-66.7)	3.3<br>(0.5-6.2)	49.2<br>(47.7 -50.7)	50.9<br>(48.9-52.8)	3.5<br>(-0.3-7.2)
Meghalaya	3,119	4,602	50.1<br>(48.4-51.9)	50.3<br>(48.8-51.7)	0.4<br>(-3.5-4.3)	56.0<br>(54.3-57.8)	58.6<br>(57.2-60.0)	4.6<br>(1.2-8.1)	58.0<br>(56.3-59.8)	58.3<br>(56.8-59.7)	0.5<br>(-2.8-3.8)	32.1<br>(30.4 -33.7)	23.9<br>(22.7-25.1)	-25.5<br>(-31.1 to -20.0)
Mizoram	3,681	1,896	56.4<br>(54.8-58.0)	52.4<br>(50.2-54.7)	-7.1<br>(-10.6 to -3.6)	78.1<br>(76.7-79.4)	79.3<br>(77.4-81.0)	1.5<br>(-0.5- 3.6)	62.8<br>(61.2-64.3)	68.1<br>(65.9-70.1)	8.4<br>(5.4-11.5)	41.5<br>(39.9 -43.1)	42.1<br>(39.9-44.4)	1.4<br>(-3.3-6.2)
Nagaland	3,136	2,205	13.5<br>(12.3-14.7)	15.6<br>(14.1-17.2)	15.6<br>(5.2-25.9)	33.8<br>(32.2-35.5)	42.5<br>(40.5-44.6)	25.7<br>(20.0-31.4)	22.6<br>(21.2-24.1)	43.2<br>(41.2-45.3)	91.2<br>(83.3-99.0)	7.6<br>(6.7- 8.6)	9.4<br>(8.3-10.7)	23.7<br>(9.3-38.1)
Odisha	9,015	7,141	64.7<br>(63.7-65.7)	80.0<br>(79.1-80.9)	23.6<br>(22.0-25.3)	83.3<br>(82.5-84.0)	90.1<br>(89.4-90.7)	8.1<br>(7.1-9.2)	77.4<br>(76.5-78.3)	92.1<br>(91.5-92.7)	19.0<br>(17.8-20.2)	47.8<br>(46.8 -48.8)	69.4<br>(68.4-70.5)	45.2<br>(42.8-47.6)
Rajasthan	11,950	10,831	38.3<br>(37.5-39.2)	56.5<br>(55.6-57.5)	47.5<br>(44.9-50.1)	85.0<br>(84.4-85.6)	95.0<br>(94.5-95.4)	11.8<br>(11.0-12.6)	65.2<br>(64.3-66.0)	85.2<br>(84.5-85.9)	30.7<br>(28.3-32.1)	29.1<br>(28.2 -29.9)	49.4<br>(48.5-50.4)	69.8<br>(66.5-73.0)
Sikkim	899	569	74.9<br>(71.9-77.6)	65.4<br>(61.4-69.2)	-12.7<br>(-17.0 to -8.3)	95.2<br>(93.6-96.4)	96.3<br>(94.4-97.5)	1.2<br>(-0.5-2.8)	75.0<br>(72.0-77.7)	73.5<br>(69.7-76.9)	-2.1<br>(-6.3-2.3)	56.0<br>(52.7-59.2)	52.0<br>(47.9-56.1)	-7.1<br>(-13.7 to -0.6)
Tripura	1,169	1,860	63.5<br>(60.7-66.2)	54.0<br>(51.7-56.2)	-15.0<br>(-19.7 to -10.3)	80.3<br>(77.9-82.5)	86.8<br>(85.2-88.3)	8.1<br>(5.1-11.1)	61.2<br>(58.4-64.0)	68.6<br>(66.4-70.7)	12.1<br>(7.2-17.0)	45.9<br>(43.0 -48.7)	39.7<br>(37.5-42.0)	-13.5<br>(-20.2 to -6.8)
Uttar Pradesh	28,741	25,556	27.4<br>(26.9-27.9)	41.5<br>(40.9-42.1)	51.5<br>(49.3-53.7)	68.1<br>(67.5-68.6)	81.1<br>(80.6-81.5)	19.1<br>(18.2-20.0)	59.1<br>(58.6 - 59.7)	76.9<br>(76.4-77.4)	30.1<br>(29.1-31.2)	20.0<br>(19.5 -20.4)	31.1<br>(30.5-31.7)	55.5<br>(52.8-58.2)
Uttarakhand	4,298	2,966	29.7<br>(28.3-31.1)	60.6<br>(58.8-62.3)	104.0<br>(98.7-109.4)	67.9<br>(66.5-69.2)	80.1<br>(78.6-81.5)	18.0<br>(15.7-20.3)	56.9<br>(55.4-58.4)	84.2<br>(82.8-85.4)	48.0<br>(45.2-50.8)	20.5<br>(19.3 -21.7)	47.7<br>(45.9-49.5)	132.7<br>(125.5-139.9)
**More developed**	**55,697**	**61,277**	**71.3<br>(70.9-71.7)**	**73.7<br>(73.4-74.1)**	**3.4<br>(2.6-4.1)**	**87.2<br>(86.9-87.5)**	**92.6<br>(92.4-92.8)**	**6.2<br>(5.8-6.6)**	**71.9<br>(71.5-72.3)**	**86.0<br>(85.7-86.3)**	**19.6<br>(19.0-20.3)**	**52.3<br>(51.9- 52.7)**	**62.5<br>(62.2-62.9)**	**19.5<br>(18.4-20.6)**
Andhra Pradesh	4,055	2,092	75.7<br>(73.9 - 77.4)	69.3<br>(67.2-71.2)	-8.5<br>(-11.0 to -5.9)	89.5<br>(88.2-90.7)	95.7<br>(94.8-96.5)	6.9<br>(5.5-8.4)	79.3<br>(77.6 – 80.9)	90.8<br>(89.5-91.9)	14.5<br>(12.3-16.7)	57.8<br>(55.7– 59.8)	62.2<br>(60.1-64.3)	7.6<br>(3.8-11.5)
Delhi	1,258	2,379	71.4	79.9	11.9	88.0	91.7	4.2	63.3	87.2	37.8	46.6	69.6	49.4

State	Sample size		Four of more ANC visits			Institutional delivery with SBA*			Post-natal care within 48 hours of delivery			Continuum of care		
	NFHS-4 (N)	NFHS-5 (N)	NFHS-4 % of N (95% CI)	NFHS-5 % of N (95% CI)	Percent change from NFHS-4 to NFHS-5 (95% CI)	NFHS-4 % of N (95% CI)	NFHS-5 % of N (95% CI)	Percent change from NFHS-4 to NFHS-5 (95% CI)	NFHS-4 % of N (95% CI)	NFHS-5 % of N (95% CI)	Percent change from NFHS-4 to NFHS-5 (95% CI)	NFHS-4 % of N (95% CI)	NFHS-5 % of N (95% CI)	Percent change from NFHS-4 to NFHS-5 (95% CI)
			(68.8 - 73.8)	(78.2-81.4)	(8.3-15.6)	(86.1-89.7)	(90.5-92.7)	(2.1-6.3)	(60.6 - 66.0)	(85.8-88.5)	(33.3-42.1)	(43.9 - 49.4)	(67.7-71.4)	(43.2-55.5)
Goa	347	322	87.6 (83.7 - 90.7)	93.2 (89.8-95.4)	6.4 (2.2-10.5)	96.8 (94.4-98.2)	98.8 (96.7-99.5)	2.1 (0.1-4.0)	91.4 (87.9 - 93.9)	96.3 (93.5-97.9)	5.4 (2.0-8.7)	80.7 (76.2 - 84.5)	90.1 (86.3-92.9)	11.6 (6.3-17.0)
Gujarat	5,827	7,575	66.4 (65.2 - 67.6)	77.7 (76.7-78.6)	17.0 (15.1-19.0)	83.9 (82.9-84.8)	90.3 (89.6-90.9)	7.6 (6.4-8.8)	62.9 (61.7 - 64.2)	89.4 (88.6-90.0)	42.1 (40.1-44.2)	42.3 (41.0- 43.5)	66.3 (65.3-67.4)	56.7 (53.5-60.0)
Haryana	5,719	5,162	45.4 (44.1 - 46.7)	60.8 (59.4-62.1)	33.9 (30.7-37.1)	82.1 (81.1-83.1)	93.4 (92.6-94.0)	13.8 (12.5-15.0)	68.3 (67.0 - 69.5)	91.3 (90.5-92.0)	33.7 (31.8-35.5)	34.3 (33.1 - 35.6)	55.6 (54.3-57.0)	62.1 (58.0-66.2)
Himachal Pradesh	2,292	2,145	66.2 (64.3 - 68.1)	71.8 (69.8-73.6)	8.5 (5.3-11.7)	75.9 (74.1-77.6)	84.3 (82.7-85.8)	11.1 (8.6-13.6)	73.6 (71.8 - 75.4)	88.9 (87.5-90.2)	20.8 (18.2-23.4)	49.5 (47.4 - 51.5)	59.3 (57.2-.61.3)	19.8 (15.2-24.4)
Jammu & Kashmir§	6,280	5,367	79.5 (78.5 - 80.5)	80.7 (79.6-81.7)	1.5 (0.1-2.9)	83.2 (82.2-84.1)	91.0 (90.2-91.7)	9.4 (8.2-10.6)	73.0 (71.9 - 74.1)	80.3 (79.2-81.3)	10.0 (8.4-11.6)	61.8 (60.6 - 63.0)	65.1 (63.8-66.4)	5.3 (3.2-7.5)
Karnataka	5,876	6,389	73.5 (72.3 - 74.6)	70.8 (69.6-71.9)	-3.7 (-5.4 to - 2.0)	90.7 (90.0-91.5)	92.5 (91.8-93.1)	2.0 (1.1-2.9)	60.5 (59.2 - 61.7)	86.4 (85.5-87.2)	42.8 (40.6-45.0)	44.6 (43.3 - 45.9)	59.1 (57.9-60.3)	32.5 (29.4-35.6)
Kerala	2,128	2,360	90.3 (89.0 - 91.5)	82.2 (80.6-83.7)	-9.0 (-10.6 to - 7.4)	99.9 (99.6-100.0)	99.8 (99.5-99.9)	-0.1 (-0.3-0.1)	86.3 (84.7 - 87.7)	92.8 (91.7-93.8)	7.5 (5.7-9.3)	78.4 (76.6 - 80.1)	77.9 (76.2-79.5)	-0.6 (-3.1-1.8)
Maharashtra	7,143	7,415	72.3 (71.3 - 73.3)	71.3 (70.3-72.3)	-1.4 (-3.0- 0.2)	86.7 (85.9-87.5)	91.0 (90.3-91.6)	5.0 (4.0-5.9)	76.9 (75.9 - 77.9)	84.1 (83.3-84.9)	9.4 (8.0-10.7)	54.4 (53.2 - 55.5)	60.2 (59.1-61.3)	10.7 (8.3-13.0)
Punjab	4,132	4,520	68.2 (66.7 - 69.6)	58.3 (56.8-59.7)	-14.5 (-16.8 to - 12.2)	92.0 (91.2-92.8)	94.1 (93.4-94.7)	2.3 (1.3-3.2)	88.4 (87.4 - 89.3)	87.1 (86.1-88.0)	-1.5 (-2.7 to -0.3)	59.1 (57.6 - 60.6)	50.2 (48.7-51.6)	-15.1 (-17.8 to -12.3)
Tamil Nadu	6,181	5,228	80.7 (79.7 - 81.7)	91.6 (90.8-92.3)	13.5 (12.2-14.8)	98.8 (98.5-99.1)	99.5 (99.3-99.7)	0.7 (0.4-1.0)	72.4 (71.3 - 73.5)	92.2 (91.4-92.9)	27.3 (25.7-29.0)	60.8 (59.6 - 62.0)	84.9 (84.0-85.9)	39.6 (37.5-41.8)
Telangana	NA	5,429	73.1 (71.0 - 75.1)	69.7 (68.5-70.9)	-4.7 (-7.6 to - 1.7)	87.2 (85.6-88.7)	91.4 (90.6-92.1)	4.8 (3.0-6.6)	81.7 (79.8 - 83.4)	86.6 (85.7-87.5)	6.0 (3.7-8.2)	57.6 (55.3– 59.9)	56.9 (55.6-58.2)	-1.2 (-5.3-2.9)
West Bengal	4,459	4,894	74.2 (72.9 - 75.5)	75.7 (74.4-76.9)	2.0 (0.1-3.9)	77.0 (75.8-78.2)	91.2 (90.4-91.9)	18.4 (16.8-20.1)	63.1 (61.7 - 64.5)	68.2 (66.9-69.5)	8.1 (5.6-10.5)	47.4 (46.0 - 48.9)	52.0 (50.6-53.4)	9.7 (6.3-13.1)

*Includes doctor, auxiliary nurse midwife, nurse, midwife, and lady health visitor

†Data missing on 101 women for institutional delivery with SBA, PNC within 48 hours of delivery, and continuum of care in NFHS-4

§Ladakh is combined with Jammu & Kashmir in NFHS-5; NFHS 4 did not provide separate data for Ladakh

Supplementary Table 3. Human Opportunity Index for geographic inequality for four of more antenatal care (ANC) visits, institutional delivery with skilled birth attendant (SBA), post-natal care within 48 hours of delivery and continuum of care for the most recent livebirth in the last 5 years in the National Family Health Survey (NFHS) rounds 4 and 5 for India and its states. CI denotes confidence interval.

	Human Opportunity Index												
	Four of more ANC visits			Institutional delivery with SBA			Post-natal care within 48 hours of delivery			Continuum of care			
	NFHS-4	NFHS-5	Percent change from NFHS 4 to NFHS-5 (95% CI)	NFHS-4	NFHS-5	Percent change from NFHS 4 to NFHS-5 (95% CI)	NFHS-4	NFHS-5	Percent change from NFHS 4 to NFHS-5 (95% CI)	NFHS-4	NFHS-5	Percent change from NFHS 4 to NFHS-5 (95% CI)
**India**	**35.8**	**48.4**	**35.2 (34.5 to 35.9)**	**68.9**	**80.4**	**16.7 (16.4 to 17.0)**	**54.6**	**73.5**	**34.6 (34.2 to 35.1)**	**24.4**	**37.1**	**52.2 (51.1 to 53.0)**
**Less developed states**	**27.9**	**40.7**	**45.9 (44.9 to 46.9)**	**64.5**	**76.2**	**18.1 (17.7 to 18.6)**	**50.6**	**69.4**	**37.2 (36.6 to 37.8)**	**18.5**	**29.7**	**60.5 (59.2 to 61.9)**
Arunachal Pradesh	20.2	34.3	69.8 (62.7 to 76.9)	47.0	77.5	64.9 (61.3 to 68.5)	22.7	51.9	128.6 (122.1 to 135.2)	8.2	21.9	167.1 (154.4 to 179.8)
Assam	40.7	45.6	12.0 (9.2 to 14.9)	66.5	82.3	23.8 (22.1 to 25.4)	51.1	64.7	26.6 (24.3 to 28.9)	24.9	31.0	24.5 (20.3 to 28.7)
Bihar	12.8	22.9	78.9 (73.9 to 83.9)	61.0	72.1	18.2 (16.8 to 19.6)	43.1	59.6	38.3 (36.2 to 40.3)	7.7	15.3	98.7 (91.8 to 105.6)
Chhattisgarh	52.0	57.2	10.0 (7.5 to 12.5)	68.6	78.5	14.4 (12.7 to 16.2)	63.0	85.3	35.4 (33.5 to 37.3)	33.1	43.3	30.8 (27.0 to 34.6)
Jharkhand	25.0	36.0	44.0 (39.7 to 48.3)	59.5	70.4	18.3 (16.4 to 20.3)	43.2	70.9	64.1 (61.4 to 66.8)	13.8	24.1	74.6 (68.3 to 81.0)
Madhya Pradesh	29.4	52.2	77.6 (74.8 to 80.3)	70.8	84.1	18.8 (17.7 to 19.8)	51.3	82.8	61.4 (59.8 to 63.0)	19.1	41.2	115.7 (111.9 to 119.5)
Manipur	54.9	61.2	11.5 (8.2 to 14.8)	58.0	62.0	6.9 (3.8 to 10.0)	54.4	57.7	6.1 (2.7 to 9.4)	39.2	41.5	5.9 (1.3 to 10.4)
Meghalaya	44.3	45.4	2.5 (-1.9 to 6.9)	49.6	52.5	5.8 (1.9 to 9.8)	52.0	53.4	2.7 (-1.1 to 6.4)	26.4	20.5	-22.3 (-28.8 to -15.9)
Mizoram	52.4	46.6	-11.1 (-14.8 to -7.3)	72.1	72.4	0.4 (-2.0 to 2.9)	59.0	63.9	8.3 (5.0 to 11.6)	37.9	36.6	-3.4 (-8.5 to 1.6)
Nagaland	8.3	11.3	36.1 (22.3 to 50.0)	26.2	37.4	42.7 (35.8 to 49.7)	17.2	37.5	118.0 (108.5 to 127.5)	4.4	6.5	47.7 (28.0 to 67.5)
Odisha	59.2	76.2	28.7 (26.8 to 30.6)	79.2	87.3	10.2 (9.1 to 11.4)	74.4	90.7	21.9 (20.6 to 23.2)	42.6	64.8	52.1 (49.4 to 54.8)
Rajasthan	33.0	51.2	55.2 (52.2 to 58.1)	81.8	93.9	14.8 (13.9 to 15.7)	61.2	82.6	35.0 (33.4 to 36.5)	24.6	44.2	79.7 (76.0 to 83.3)
Sikkim	71.3	60.1	-15.7 (-20.5 to -10.9)	94.4	95.2	0.8 (-0.9 to 2.6)	71.9	70.4	-2.1 (-6.7 to 2.5)	52.1	48.3	-7.3 (-14.4 to -0.2)
Tripura	60.4	50.4	-16.6 (-21.6 to -11.5)	76.4	83.5	9.3 (5.9 to 12.7)	58.2	64.2	10.3 (5.1 to 15.5)	42.9	35.3	-17.7 (-24.8 to -10.6)
Uttar Pradesh	21.9	38.1	74.0 (71.4 to 76.6)	64.0	78.0	21.9 (20.9 to 22.8)	53.5	73.2	36.8 (35.6 to 38.0)	15.6	27.9	78.8 (75.6 to 82.1)
Uttarakhand	25.9	55.7	115.1 (109.0 to 121.1)	65.2	78.5	20.4 (18.0 to 22.8)	54.3	81.5	50.1 (47.1 to 53.1)	17.5	43.3	147.4 (139.3 to 155.5)
**More developed states**	**65.2**	**68.2**	**4.6 (3.9 to 5.3)**	**82.8**	**90.1**	**8.8 (8.4 to 9.2)**	**66.2**	**82.6**	**24.8 (24.1 to 25.4)**	**45.7**	**56.3**	**23.2 (22.2 to 24.2)**
Andhra Pradesh	74.3	68.3	-8.1 (-10.8 to -5.4)	88.0	95.4	8.4 (6.8 to 10.0)	77.6	90.4	16.5 (14.2 to 18.8)	56.1	59.3	5.7 (1.7 to 9.7)
Goa	86.0	92.4	7.4 (3.0 to 11.9)	96.7	98.5	1.9 (-0.2 to 3.9)	90.5	95.6	5.6 (2.1 to 9.2)	79.0	88.8	12.4 (6.7 to 18.1)
Gujarat	59.7	73.1	22.4 (20.2 to 24.7)	80.1	87.5	9.2 (7.9 to 10.6)	59.9	87.1	45.4 (43.2 to 47.6)	36.7	61.2	66.8 (63.1 to 70.4)
Haryana	37.3	56.6	51.7 (47.9 to 55.5)	75.6	90.5	19.7 (18.1 to 21.3)	61.3	88.7	44.7 (42.5 to 46.9)	27.2	51.2	88.2 (83.3 to 93.1)
Himachal Pradesh	61.7	67.1	8.8 (5.2 to 12.3)	72.7	82.5	13.5 (10.8 to 16.2)	69.8	87.3	25.1 (22.2 to 27.9)	45.4	54.3	19.6 (14.6 to 24.6)
Jammu & Kashmir*	71.9	76.3	6.1 (4.4 to 7.8)	77.8	87.9	13.0 (11.6 to 14.4)	67.1	77.2	15.1 (13.2 to 16.9)	53.3	59.5	11.6 (9.1 to 14.2)
Karnataka	69.3	65.1	-6.1(-7.9 to -4.2)	88.4	90.3	2.1 (1.1 to 3.1)	55.3	83.6	51.2 (48.8 to 53.6)	39.6	52.6	32.8 (29.4 to 36.3)
Kerala	88.9	77.3	-13.0 (-14.8 to -11.3)	99.8	99.7	-0.1 (-0.3 to 0.1)	84.2	91.5	8.7 (6.7 to 10.6)	75.7	72.9	-3.7 (-6.3 to -1.1)
Maharashtra	69.3	66.7	-3.8(-5.5 to -2.0)	83.9	88.2	5.1 (4.0 to 6.2)	74.2	80.8	8.9 (7.4 to 10.4)	50.0	54.5	9.0 (6.4 to 11.6)
Delhi	68.8	75.3	9.4 (5.5 to 13.3)	86.0	90.2	4.9 (2.6 to 7.2)	59.1	85.1	44.0 (39.3 to 48.7)	42.7	65.3	52.9 (46.2 to 59.7)
Punjab	64.6	54.7	-15.3 (-17.8 to -12.8)	91.1	92.9	2.0 (1.0 to 3.0)	86.9	85.4	-1.7 (-3.0 to -0.4)	55.5	46.9	-15.5 (-18.5 to -12.5)
Tamil Nadu	77.5	89.6	15.6 (14.2 to 17.0)	98.4	99.2	0.8 (0.5 to 1.1)	68.4	90.4	32.2 (30.4 to 33.9)	56.4	82.0	45.4 (43.0 to 47.7)
Telangana	71.8	68.2	-5.0 (-8.0 to -2.0)	85.6	90.4	5.6 (3.7 to 7.6)	79.9	85.4	6.9 (4.5 to 9.3)	55.7	54.9	-1.4 (-5.7 to 2.8)
West Bengal	69.4	72.2	4.0 (1.9 to 6.2)	71.3	89.2	25.1 (23.2 to 27.1)	58.0	63.8	10.7 (7.3 to 12.7)	42.0	47.3	12.6 (8.8 to 16.4)

*Ladakh is combined with Jammu & Kashmir in NFHS-5; NFHS 4 did not provide separate data for Ladakh.

Supplementary Table 4. Ratio of Human opportunity index (HOI) between wealth index quintiles I and V for the maternal services for India and its states, National Family Health Survey-5.

	Ratio of HOI between wealth index quintiles I and V			
	Four of more antenatal care visits	Institutional delivery with skilled birth attendant	Post-natal care within 48 hours of delivery	Continuum of care
**India**	0.52	0.69	0.73	0.38
**Less developed states**	0.55	0.69	0.74	0.41
Arunachal Pradesh	0.43	0.58	0.70	0.38
Assam	0.63	0.75	0.79	0.52
Bihar	0.32	0.72	0.71	0.25
Chhattisgarh	0.89	0.72	0.93	0.70
Jharkhand	0.51	0.64	0.81	0.36
Madhya Pradesh	0.72	0.83	0.91	0.60
Manipur	0.40	0.40	0.48	0.30
Meghalaya	0.66	0.47	0.88	0.50
Mizoram	0.16	0.28	0.36	0.12
Nagaland	0.27	0.35	0.47	0.06
Odisha	0.84	0.81	0.94	0.69
Rajasthan	0.77	0.92	0.92	0.73
Sikkim	1.41	0.94	1.17	1.27
Tripura	0.81	0.79	0.68	0.38
Uttar Pradesh	0.60	0.76	0.80	0.44
Uttarakhand	0.51	0.68	0.81	0.41
**More developed states**	0.83	0.80	0.76	0.63
Andhra Pradesh	0.66	0.75	0.73	0.50
Goa	1.07	1.01	1.01	1.09
Gujarat	0.79	0.83	0.96	0.71
Haryana	0.52	0.67	0.70	0.39
Himachal Pradesh	0.70	0.60	0.77	0.49
Jammu and Kashmir	0.76	0.74	0.78	0.61
Karnataka	0.75	0.90	0.91	0.64
Kerala	0.95	1.00	0.89	0.95
Maharashtra	0.68	0.73	0.78	0.51
Delhi	0.34	0.24	0.36	0.02
Punjab	0.54	0.84	0.80	0.57
Tamil Nadu	1.01	0.96	0.94	0.96
Telangana	0.78	0.83	0.89	0.63
West Bengal	0.85	0.90	0.70	0.57

Ratio of HOI

(red)	Less than 0.50
(orange)	0.51-0.89
(light yellow)	0.90-1.09
(blue)	1.10-1.49
(green)	1.50 or more
