# Supplementary material for: Population-level trends over a decade in geographical inequality for opportunity in access to maternal care services: a cross-sectional analysis from the National Family Health Surveys in India
Source: BMJ Open. 2024 Nov 21;14(11):e083922. doi: 10.1136/bmjopen-2024-083922 (PMC11590786; doi:10.1136/bmjopen-2024-083922)
Supplement: Supplementary file 2 [file bmjopen-2024-083922.reviewer_comments.pdf]

PEER REVIEW HISTORY

BMJ Open publishes all reviews undertaken for accepted manuscripts. Reviewers are asked to complete a checklist review form and are provided with free text boxes to elaborate on their assessment. These free text comments are reproduced below.

ARTICLE DETAILS

Title (Provisional)

Population-level trends over a decade in geographical inequality for opportunity in access to maternal care services: a cross-sectional analysis from the National Family Health Surveys in India

Authors

Dandona, Rakhi; Majumder, Moutushi; Kumar, G Anil

VERSION 1 - REVIEW

**Reviewer**	**1**
**Name**	**Tounkara, Moctar**
**Affiliation**	**USTTB**
**Date**	**14-Feb-2024**
**COI**	**I have not**

The manuscript contains many abbreviations which were not defined during their first appearance.

the author talks about geographic inequity and the data analysis was carried out with socio-economic data and particularly the economic well-being index.

**Reviewer**	**2**
**Name**	**Farooq, Fouzia**
**Affiliation**	**The George Washington University, Department of Global Health**
**Date**	**21-Feb-2024**
**COI**	**NA**

The paper references different supplementary tables mixed with main paper tables which makes it difficult to follow. I suggest revising the paper so each section follows a clear methodology.

Authors mentioned the use of PCA for wealth index. Did the authors employ the PCA themselves or they are just mentioning that NFHS used PCA to calculate WI. It is unclear from the methods.

There are way too many acronyms here. Unless they are very commonly used, please write out the phrases.

I also suggest making the results more visual with figures.

Trends over a decade in geographical inequality for opportunity in access to maternal care services from the National Family Health Surveys in India

Dandona et al., 2024 (Anil Kumar group) at Public Health Foundation of India + Institute of Health metrics and Evaluation, University of Washington, Seattle.
BMJ Open

Abstract:

1. Line 8 – Please write out ENAP in the abstract.

Introduction:

2. Lines 21-23 – What does timeline of "between 2011 and 2015-2016" mean?

Methods:

3. Lines 97-98 do not belong under the heading 'data analysis'.
4. Lines 110 – belongs in the results section.
5. Line 106 – can be moved up and combined with line 102. Describe your definition of "D" when you are describing this measure in line 102.
6. Please discuss NFHS's sampling methods.

Results:

1. Would be great to see a Table 1 showing the distribution of these services based on baseline factors such as age, education level, religion etc.
2. Lines 136-139 are a bit unclear. Perhaps break it up so you list the estimate for India overall first. Then list the ones for less developed and more developed.
3. Heading in Table 1 needs to be clear that it is 'HOI by WI quintiles'. Please clarify headings such as "WI I: average" – is this HOI average?
4. Throughout the results section, authors list numbers as "in India, more developed and less developed states in NSFH-X, respectively). Perhaps rephrase these sentences because they're very unclear.
5. I am also not sure what adding information like "X% in Nagaland vs. Y% in Odisha in less developed states" is conveying. I suggest rephrasing so that the message is highlighted, such as: "this coverage ranged from X% - Y% in less developed states".
6. Suggest showing a table of sample size of different services being utilized such as ANC, Skill attendant birth etc in both NFHS-4 and NFHS-5.
7. Suggest showing some of these data in figures such as line graph or scatter plot for different states/regions.

Overall comments: The paper references different supplementary tables mixed with main paper tables which makes it difficult to follow. I suggest revising the paper so each section follows a clear methodology. Authors mentioned the use of PCA for wealth index. Did the authors employ the PCA themselves or they are just mentioning that NFHS used PCA to calculate WI. It is unclear from the methods.
There are way too many acronyms here. Unless they are very commonly used, please write out the phrases.
I also suggest making the results more visual with figures.

**Reviewer**	**3**
**Name**	**Okoli, Chijioke**
**Affiliation**	**University of Southern Queensland, School of Business**
**Date**	**08-May-2024**
**COI**	**None.**

BMJ Open **manuscript review**

bmjopen-2024-083922

Trends over a decade in geographical inequality for opportunity in access to maternal care services from the National Family Health Surveys in India.

Reviewer's comment:

The authors have examined district-level inequality in India and its states with regards to achieving Every Newborn Action Plan (ENAP) 2025 coverage targets. This research is vital given that the Sustainable Development Goals of 3.1 and 3.2 targets by 2030 are around the corner.

However, the authors may consider the following comments below to strengthen the manuscript.

Abstract:

In lines 28-30, the statement under primary and secondary measures subheading "District-level coverages of 4+ antenatal care (ANC) visits, institutional delivery with skilled birth attendant (SBA), postnatal care (PNC) services within 48 hours of delivery, Continuum of care (CoC) services for women with most recent livebirths" **is an incomplete sentence.**

Replace "primary and secondary measures" with "methods" to align with what is in the body of the manuscript.

State the type of data used (primary and/or secondary) and total of number of study participants.

Methods:

Provide a brief profile of the study setting before the study design. The subheading could be "Study setting."

In lines 90-91, under the study design, provide the total number of livebirths for both datasets.

In lines 123-124, explain why all the other Union Territories were excluded in the study.

Results:

In lines 141-144, "The HOI for geographic inequality in 4+ ANC visits for India was 48.4 in NFHS-5, an improvement of 35.2% (95% CI 34.5 to 35.9) between NFHS-4 to NFHS-5 (supplemental table 2) and was substantially higher for the more developed states at 68.2 than the less developed states at 40.7 in NFHS-5 (table 1 and supplemental table 3)". Put percent (%) sign e.g., 48.4%, 68.2% and 40.7%. Also, check the rest of the manuscript to put the sign where necessary.

In lines 135, 165, and 191 write in full ANC, SBA, and PNC and put the acronym in bracket, respectively.

Discussion:

In line 304, delete “dyad” or make a necessary correction.

Conclusion:

In lines 328 – 330, break the long sentence into two and rewrite to convey the intended message.

General comment:

Proofreading is highly recommended.

VERSION 1 - AUTHOR RESPONSE

Reviewer: 1

8.The manuscript contains many abbreviations which were not defined during their first appearance.

We have checked the entire manuscript for abbreviations. We have written in full all the abbreviations when these appear the first time.

9. The author talks about geographic inequity and the data analysis was carried out with socio-economic data and particularly the economic well-being index.

The primary analysis presented in this manuscript is geographical inequality defined at the district-level. We have now clearly mentioned this in lines 80-83. With this geographic inequality, we also report inequality by wealth index.

Reviewer: 2

10. Line 8 – Please write out ENAP in the abstract.

We have updated the full form of ENAP in line number 26.

11. Lines 21-23 – What does timeline of “between 2011 and 2015-2016” mean?

We have clarified this now in lines 92 – 95.

12. Lines 97-98 do not belong under the heading “data analyses”.

We have now re-phrased line 101.

13. Lines 110 – belongs in the results section.

We have stated the range of HOI value here (0 to 100). This is not a result.

14. Line 106 – can be moved up and combined with line 102. Describe your definition of "D" when you are describing this measure in line 102.

Thank you for the suggestion. We have now moved the text as suggested (lines 104-107).

15. Please discuss NFHS's sampling methods.

The details of study setting for NFHS are already provided in lines 86-90. These are well published surveys and appropriate references have been provided.

16. Would be great to see a Table 1 showing the distribution of these services based on baseline factors such as age, education level, religion etc.

The aim of this paper is to present geographic inequity in the maternal health services. Hence, the suggestion is beyond the scope of this manuscript. The NFHS reports are available in public domain that provide these details, and are referenced in this manuscript.

17. Lines 136-139 are a bit unclear. Perhaps break it up so you list the estimate for India overall first. Then list the ones for less developed and more developed.

Thank you for this suggestion. We have updated lines 143-144 as suggested, and also undertaken this change in lines 171-173, 194-196, and 218-220.

18. Heading in Table 1 needs to be clear that it is 'HOI by WI quintiles'. Please clarify headings such as "WI I: average" – is this HOI average?

We have updated the headers Tables 1, 2, 3 and 4, respectively.

19. Throughout the results section, authors list numbers as "in India, more developed and less developed states in NSFH-X, respectively). Perhaps rephrase these sentences because they're very unclear.

As indicated above, we have updated these everywhere.

20. I am also not sure what adding information like "X% in Nagaland vs. Y% in Odisha in less developed states" is conveying. I suggest rephrasing so that the message is highlighted, such as: "this coverage ranged from X% - Y% in less developed states".

We have provided coverage range by the type of states (less and more developed), and also given some states as relevant examples in certain places. It is important to showcase the range within the less and more developed states also in addition to between these two types of states.

21. Suggest showing a table of sample size of different services being utilized such as ANC, Skill attendant birth etc in both NFHS-4 and NFHS-5.

We have now added the sample size for NFHS-4 and NFHS-5 in the Supplementary Table 2. It is not possible to add number of women utilising each maternal service for each state and for two surveys as the table will get unwieldy. This can now be easily calculated based on the percent provided for each maternal service and the sample size in each state which is now added.

22. Suggest showing some of these data in figures such as line graph or scatter plot for different states/regions.

Thank you for this suggestion. However, we believe that India maps are the best way to represent the geographic inequity in this analysis as combining indicators across scatter plot with nearly 30 states will be unwieldy for interpretation.

23. The paper references different supplementary tables mixed with main paper tables which makes it difficult to follow. I suggest revising the paper so each section follows a clear methodology.

Like any other manuscript, there is a mix of main tables and supplementary tables. These are indicated in the sequence to the relevance of text reported. We have reviewed the methods section again in light of this comments. We believe that it is clear in reporting of the datasets and the analysis undertaken.

24. Authors mentioned the use of PCA for wealth index. Did the authors employ the PCA themselves or they are just mentioning that NFHS used PCA to calculate WI? It is unclear from the methods.

As stated in lines 116-119, wealth Index derived from PCA technique is already available in the NFHS dataset. We did not have to calculate it.

25.There are way too many acronyms here. Unless they are very commonly used, please write out the phrases.

We understand. We leave this to the Editor to decide.

26. I also suggest making the results more visual with figures.

Thank you. We reviewed the figures currently in the manuscript. We have provided figures for all the major results but under one Figure. We have now split the Figures into 4 (Figures 1 to 4) which show the visual representation of HOI for 4+ antenatal care visits, institutional delivery with skilled birth attendant, post-natal care in 48 hours of delivery, and continuum of care, respectively.

Reviewer: 3

27**.** The authors have examined district-level inequality in India and its states with regards to achieving Every Newborn Action Plan (ENAP) 2025 coverage targets. This research is vital given that the Sustainable Development Goals of 3.1 and 3.2 targets by 2030 are around the corner. However, the authors may consider the following comments below to strengthen the manuscript.

Thank you.

28. In lines 28-30, the statement under primary and secondary measures subheading “District-level coverages of 4+ antenatal care (ANC) visits, institutional delivery with skilled birth attendant (SBA), postnatal care (PNC) services within 48 hours of delivery, Continuum of care (CoC) services for women with most recent livebirths” is an incomplete sentence.

Response: Thank you, we have now updated this sentence in line number 32-33.

29. Replace "primary and secondary measures" with "methods" to align with what is in the body of the manuscript.

The "primary and secondary measures" are as per the BMJ Open's abstract format.

30. State the type of data used (primary and/or secondary) and total of number of study participants.

We have used the publicly available data for this analysis as stated in lines 136-137. The total number of study participants are given.

31. Provide a brief profile of the study setting before the study design. The subheading could be "Study setting."

We have deleted the sub-heading of "Study design" as it was not appropriate. The details of study setting are already provided in lines 86-90.

32. In lines 90-91, under the study design, provide the total number of livebirths for both datasets.

We have now added the total number of livebirths in lines 136-137.

33. In lines 123-124, explain why all the other Union Territories were excluded in the study.

The other Union Territories were excluded as there are no districts in these Union Territories to undertake this analysis. We have now stated this in lines 127-129.

34. In lines 141-144, "The HOI for geographic inequality in 4+ ANC visits for India was 48.4 in NFHS-5, an improvement of 35.2% (95% CI 34.5 to 35.9) between NFHS-4 to NFHS-5 (supplemental table 2) and was substantially higher for the more developed states at 68.2 than the less developed states at 40.7 in NFHS-5 (table 1 and supplemental table 3)". Put percent (%) sign e.g., 48.4%, 68.2% and 40.7%. Also, check the rest of the manuscript to put the sign where necessary.

HOI is an index which ranges from 0-100 where 0 (high inequality) and 100 (universal access). This is stated in lines 109-111. Therefore, percent sign is not applicable to HOI.

35. In lines 135, 165, and 191 write in full ANC, SBA, and PNC and put the acronym in bracket, respectively.

All the abbreviations have been written in full when cited for the first time in the manuscript.

36. In line 304, delete "dyad" or make a necessary correction.

Dyad is appropriate here as we are referring to mother-child pair.

37. In lines 328 – 330, break the long sentence into two and rewrite to convey the intended

message.

We have now split this into two sentences (lines 313-334).

38. Proofreading is highly recommended.

The manuscript has been re-read and necessary corrections have been incorporated.

Thank you for considering our manuscript. If more information were needed, I would be pleased to respond.

Best regards,

Rakhi Dandona

VERSION 2 - AUTHOR RESPONSE
